# Seasonal and Regional Dynamics of *M. ulcerans* Transmission in Environmental Context: Deciphering the Role of Water Bugs as Hosts and Vectors

**DOI:** 10.1371/journal.pntd.0000731

**Published:** 2010-07-06

**Authors:** Estelle Marion, Sara Eyangoh, Edouard Yeramian, Julien Doannio, Jordi Landier, Jacques Aubry, Arnaud Fontanet, Christophe Rogier, Viviane Cassisa, Jane Cottin, Agnès Marot, Matthieu Eveillard, Yannick Kamdem, Pierre Legras, Caroline Deshayes, Jean-Paul Saint-André, Laurent Marsollier

**Affiliations:** 1 Groupe d'Etude des Interactions Hôte-Pathogène, Université d'Angers, Angers, France; 2 Laboratoire des Mycobactéries, Centre Pasteur du Cameroun, Yaoundé, Cameroun; 3 Unité de Bio-Informatique Structurale, Institut Pasteur, Paris, France; 4 Institut National de Santé Publique, Abidjan, Côte d'Ivoire; 5 Unité d'Epidémiologie des Maladies Emergentes, Institut Pasteur, Paris, France; 6 INSERM U601, Université de Nantes, Faculté de Pharmacie, Nantes, France; 7 Unité de Recherche sur les Maladies Infectieuses et Tropicales Emergentes, UMR6236, Institut de Recherche Biomédicale des Armées, Marseille, France; 8 Laboratoire de Bactériologie, Centre Hospitalier Universitaire, Angers, France; 9 Animalerie Hospitalo-Universitaire, Angers, France; 10 Laboratoire d'Anatomie Pathologique et Faculté de Médecine, CHU, Angers, France; Institut Pasteur, France

## Abstract

**Background:**

Buruli ulcer, the third mycobacterial disease after tuberculosis and leprosy, is caused by the environmental mycobacterium *M. ulcerans*. Various modes of transmission have been suspected for this disease, with no general consensus acceptance for any of them up to now. Since laboratory models demonstrated the ability of water bugs to transmit *M. ulcerans*, a particular attention is focused on the transmission of the bacilli by water bugs as hosts and vectors. However, it is only through detailed knowledge of the biodiversity and ecology of water bugs that the importance of this mode of transmission can be fully assessed. It is the objective of the work here to decipher the role of water bugs in *M. ulcerans* ecology and transmission, based on large-scale field studies.

**Methodology/Principal Findings:**

The distribution of *M. ulcerans*-hosting water bugs was monitored on previously unprecedented time and space scales: a total of 7,407 water bugs, belonging to large number of different families, were collected over one year, in Buruli ulcer endemic and non endemic areas in central Cameroon. This study demonstrated the presence of *M. ulcerans* in insect saliva. In addition, the field results provided a full picture of the ecology of transmission in terms of biodiversity and detailed specification of seasonal and regional dynamics, with large temporal heterogeneity in the insect tissue colonization rate and detection of *M. ulcerans* only in water bug tissues collected in Buruli ulcer endemic areas.

**Conclusion/Significance:**

The large-scale detection of bacilli in saliva of biting water bugs gives enhanced weight to their role in *M. ulcerans* transmission. On practical grounds, beyond the ecological interest, the results concerning seasonal and regional dynamics can provide an efficient tool in the hands of sanitary authorities to monitor environmental risks associated with Buruli ulcer.

## Author Summary

Buruli ulcer, caused by *Mycobacterium ulcerans*, is a devastating skin disease. Most cases of Buruli ulcer occur in poor rural communities. As a result, treatment is frequently sought too late and about 25% of those infected—particularly children—become permanently disabled. Outbreaks of Buruli ulcer have always been associated with swampy areas. However, the route(s) of bacillus transmission is (are) still unclear. This *Mycobacterium* species resides in water where it colonizes many ecological niches such as aquatic plants, herbivorous animals and predatory/carnivorous insects. For several years the role of water bugs as hosts and vectors of *M. ulcerans* was suspected and was demonstrated under laboratory conditions. The aim of this work was to further assess the role of water bugs as hosts and vectors of *M. ulcerans* in environmental context. This work identifies several water bug families as hosts of *M. ulcerans* in Buruli ulcer endemic area. The detection of bacilli in saliva of human biting insects provides further evidence for their role in *M. ulcerans* transmission. Interestingly, three of these insects are good flyers, and as such could participate in *M. ulcerans* dissemination.

## Introduction

*Mycobacterium ulcerans* is the causative agent of Buruli ulcer. This devastating necrotic human skin disease is the third most common mycobacterial disease, after tuberculosis and leprosy. The detection rates of Buruli ulcer were found to increase gradually and steadily. The range of the disease extends from 10°N to 10°S latitude in Africa and spans 16 endemic countries, 10 potential endemic countries and 20 non endemic countries. The majority of cases are localized in Africa, with cases also reported in Asia, Australia and South America. In Africa, Buruli ulcer occurs mainly in poor rural communities [1]–[5]. As a consequence, very often treatment is sought and prescribed too late. Treatment of later stages requires extensive surgery at major hospitals, involving prolonged and very expensive stays, with 25% of those who experienced Buruli ulcer -in particular children- becoming permanently disabled [6]–[7]. More recently, it was possible to cure the disease at an early stage with antibiotics, without the need for surgery. The administration of a combination of rifampin and an aminoglycoside for four to eight weeks led to the healing of early lesions, without radical surgery. This antibiotics-based treatment is now the recommended standard regimen in areas where information about Buruli ulcer was made accessible, resulting in early diagnosis of the disease [8]–[9].

In 1998, the World Health Organization launched the Global Buruli Ulcer Initiative to intensify surveillance, disease control, treatment and also to promote understanding of the ecology and the mode of transmission of *M. ulcerans*.

There is at present no clear understanding of the exact mode(s) of transmission of *M. ulcerans*. Populations affected by Buruli ulcer are those living close to humid and swampy zones. Indeed the foci of the disease are associated with the creation or the extension of swampy areas, such as construction of dams or lakes for the development of agriculture (irrigation) [2]–[3], [10]–[17].

Over the last few decades, different mechanisms have been proposed for the transmission of *M. ulcerans* from aquatic environments to human skin, ranging from aerosol contamination (an hypothesis invoked in Australia but never confirmed) [12] to insect-dependent transmission. The role of insects is indeed suggested by various studies over the past ten years. Recently, *M. ulcerans* DNA was detected in 0.04% of mosquito populations [18]–[20]. One possible transmission route is through the subversion of *Aedes* and *Anopheles* aquatic larvae (inhabiting *M. ulcerans*-loaded water) as hosts, with the human blood-feeding adults delivering *M. ulcerans* in the skin. However at present this hypothesis has not been confirmed experimentally, with no detection of *M. ulcerans* in the saliva or salivary glands. As the blood-feeding adults emerging from aquatic larvae cannot account for *M. ulcerans* transmission in any general way, different investigators have proposed that biting water bugs could act as vectors for *M. ulcerans*. In 1999, Portaels was first to raise this hypothesis, and also to isolate *M. ulcerans* from water bug tissues [21]–[22]. Our pioneering experimental, laboratory-based, studies on mice have given weight to this hypothesis. These studies showed that *M. ulcerans* was able to colonize the salivary glands of water bugs, which could then transmit *M. ulcerans* to mice through biting [23]–[28]. Other field investigations allowed detecting *M. ulcerans* DNA in the water bugs captured in Buruli ulcer endemic areas [21], [28]–[30]. On the other hand, a field study conducted in Ghana [30] did not support the role of biting Hemiptera or other invertebrates as possible *M. ulcerans* hosts/reservoirs or vectors. This study rather pointed out the need for further research to better understand *M. ulcerans* transmission [31]. In this context, our first study on the ability of water bugs to transmit bacterium through biting was confirmed recently in an invertebrate model [32].

Water bugs are familiar insects in aquatic habitats throughout the world. They are present in all Buruli ulcer endemic areas. They belong to the order of Hemiptera, containing several families. There are basically two kinds of water bugs: the semi-aquatic bugs, which live upon the water surface and the true water bugs, which live beneath the water surface. These invertebrates live in a wide variety of natural habitats, lakes and rivers ranging from small to large and also small ponds. Most water bugs can be characterized as predatory feeders, preying on aquatic invertebrates (insect larvae, snails, etc.) [33]. Water bugs can also feed on small vertebrates such as fishes and amphibians. In addition, a water bug family was reported to feed on plant material [33]. Most water bug species are able to fly, flying mainly at night when attracted by light, a feature that could account for *M. ulcerans* dissemination in the environment as suggested by Portaels and Meyers [34]. In this direction, *M. ulcerans* DNA was detected recently in water bugs collected out of their aquatic environment, thus demonstrating their flying capacity (Marsollier, personal communication).

In tropical areas, water bug biodiversity and biology are poorly documented, making it difficult (i) to define their role as *M. ulcerans* hosts and vectors and (ii) to characterize the relations between *M. ulcerans* and these aquatic insects.

In this context, the present investigations had three objectives:

1. To establish an inventory of water bug genera and species in central Cameroon, comparing Buruli ulcer endemic and non endemic areas.
2. To investigate the water bug population dynamics throughout the seasons.
3. To monitor the presence of *M. ulcerans* DNA i) not only in bodies of water bugs from the inventoried family but also ii) in the saliva collected from these water bugs during the four seasonal periods. Of note, when the *M. ulcerans* DNA positive saliva was inoculated in the mouse tail skin, lesions displaying features of Buruli ulcer did develop.

## Materials and Methods

### Sites of study

**Figure 1:**
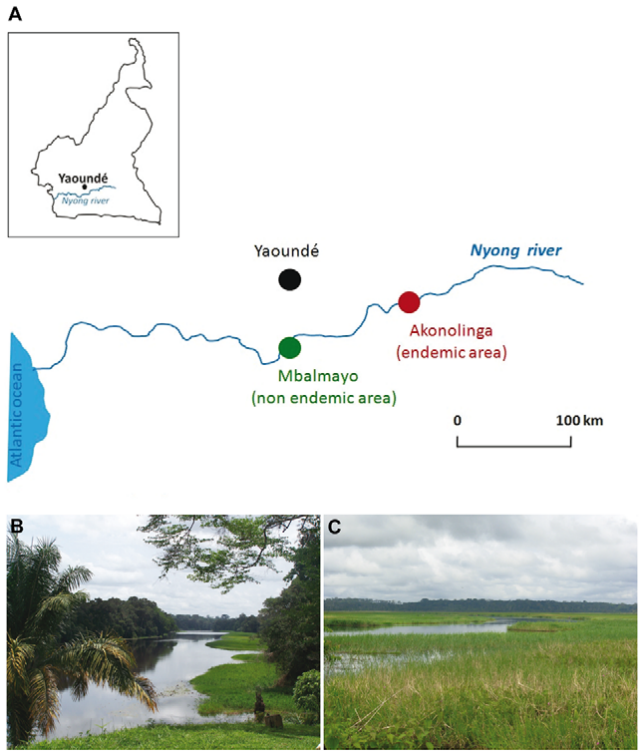
Locations of sampling sites along the Nyong River (Cameroon). (A) The samples of aquatic insects were collected on the Nyong river in an endemic area (Akonolinga) and in a non endemic area (Mbalmayo) for Buruli ulcer. In the non endemic site, the bank of the Nyong (B) harbours a dense evergreen forest contrary to the endemic sites with an open vegetation landscape (C) caused by intense agricultural activities.

Regular sampling of aquatic insects was performed between October 2007 and July 2008, in two areas of the Centre Province of Cameroon: a Buruli ulcer endemic area in the Nyong River basin (Akonolinga, 3.77334N 12.24135E) and a Buruli ulcer non endemic area (Mbalmayo, 3.51552N 11.50085E) situated 100 km downstream (Figure 1). The two areas, along the Nyong River, were selected on the basis of their accessibility all year long (i.e. including in rainy season) and of the availability of relevant epidemiological studies. Prevalence of Buruli ulcer endemic site (Akonolinga district) is estimated at 0.47% [16], [35]–[36] and no case of Buruli ulcer has been reported at this day in Mbalmoyo. Both populations are primarily involved in fishing, which is supplemented by riverbank agriculture in the endemic site. The population densities are 21 h/km^2^ and 40 h/km^2^ in the endemic and non-endemic sites, respectively. Six and two large sampling collections were carried out respectively in the endemic site (October, November, December, January, April and July) and in the non-endemic site (April and July). Water bodies were sampled from the main water sources: domestic washing, bathing, fishing and recreational sources. It is also noticeable that the sites under study corresponded to meeting points for the population, to cross the Nyong River in dugout canoes.

### Aquatic insect capture, sampling and pooling

Sampling was conducted between 8:00 am and 12:00 noon for all sites, with the same sampling methods. As aquatic bugs are associated in general with aquatic plants, the exploration was restricted to this ecological niche, along the bank of the Nyong River. In order to minimize escape of insects, a canoe was used to access the capture site. The insects were captured with a square-net (32×32 cm and 1 mm in mesh size) from the surface to a depth of 1 meter, and over a distance of 1 meter. Each month, 5 samplings were performed on each of 3 consecutive mornings. A given sample corresponds to the mix of all insects collected after 10 sweeps. All insects were preserved in 70% ethanol for laboratory identification. The adults as well as nymph insects were numbered individually for taxa identification. To detect *M. ulcerans* DNA, the insects were sorted into pooled groups, including fewer than 10 specimens from the same family.

### Water bug family identification

The water bugs were classified in phylum Arthropoda, class Insecta, order Hemiptera and suborder Heteroptera. The main criteria for Heteroptera identification were as follows :

- piercing-sucking mouthparts, with a segmented rostrum arising from the front of the head
- two pairs of wings in adults: partly membranous forewings -hemelytra- and fully membranous hind wings

The classification of the collected samples into families was performed based on the application of the heteroptera family determination criteria to each specimen [37].

### Insect saliva collection

Additional Belostomatidae (*Appasus sp.*) were selectively gathered by sweep sampling. They were transported to the laboratory in plastic containers with an air pump (Pafex 3 aerator, Pafex) in fresh water. It should be noted that these collected insects were not included in the counting of water bugs to study variation of water bug density and detection of *M. ulcerans* in water bug tissues. The saliva was collected by a method similar to that used for mosquito saliva [38]–[39], with appropriate modifications. As a difference to the case of mosquito salivation, there was no need to remove legs and wings in order to sedate the insect and to inoculate a solution in the thorax region. The body of the water bug was grasped with metallic blunt pincers and its rostrum was placed into a conventional plastic pipette tip containing 10 µl of sterile water. With such manipulation white saliva fluid could be observed after 2 minutes time. From each individual saliva sample, 5 µl were used for quantitative PCR and 5 µl kept for Ziehl-Neelsen staining and mouse tail inoculation experiments in PCR positive cases (Figure S1).

### DNA extraction from water bug tissues and saliva

Pooled insect bodies were ground and homogenized in 50 mM NaOH solution. Tissue homogenates were heated at 95°C for 20 min. The samples were neutralized by 100 mM Tris-HCl, pH 8.0. DNA from homogenized insect tissues was purified using Power Soil DNA isolation kit (MO Bio lab, Carlsbad, CA), according to manufacturer's recommendations. 5 µl of each individual saliva sample was resuspended in 50 µl of 50 mM NaOH, heated and neutralized as described above. No purification was needed because no PCR inhibitor was present after DNA extraction from saliva. To eliminate DNA traces after each extraction, homogenizers were decontaminated overnight in 1 M NaOH and rinsed in distilled water before sterilization at 130°C for 20 min.

### Detection of *M. ulcerans* DNA by quantitative PCR

Oligonucleotide primer and TaqMan probe sequences were selected from the GenBank IS*2404* sequence [40] and the ketoreductase B (KR) domain of the mycolactone polyketide synthase (mls) gene (Table 1) from the plasmid pMUM001 [40]–[43]. PCR mixtures contained 5 µl of template DNA, 0.3 µM concentration of each primer, 0.25 µM concentration of the probe, and IQSupermix (Bio-Rad Lab) in a total volume of 25 µl. Amplification and detection were performed with Thermocycler (MX3000P, Stratagene) using the following program: 1 cycle of 50°C for 2 min, 1 cycle of 95°C for 15 min, 40 cycles of 95°C for 15 s and 60°C for 1 min. DNA extracts were tested at least as duplicates, and negative controls were included in each assay. Quantitative readout assays were set up, based on external standard curve with *M. ulcerans* (strain 1G897) DNA serially diluted over 8 logs. Samples were considered positive only if both IS*2404* sequence and the gene sequence encoding the ketoreductase B domain (KR) of the mycolactone polyketide synthase were detected, with threshold cycle (Ct) values strictly <35 cycles.

**Table 1:** Primers and probes used to detect *M. ulcerans* DNA sequences by Taq Man real-time PCR.

Primer or Probe Name	Sequence (5′ to 3′)
IS*2404* forward primer	ATTGGTGCCGATCGAGTTG
IS*2404* reverse primer	TCGCTTTGGCGCGTAAA
IS*2404* probe	FAM-CACCACGCAGCATTCTTGCCGT-TAMRA
KR-B forward primer	TCACGGCCTGCGATATCA
KR-B reverse primer	TTGTGTGGGCACTGAATTGAC
KR-B probe	FAM-ACCCCGAAGCACTGGCCGC-TAMRA

### Quality control and quality assurance of clinical and environmental diagnostic PCR

The university laboratory is enrolled in quality control of clinical specimens, along with three partners: Angers University Hospital, Pasteur Centre of Yaoundé (Cameroon), and Institut Pasteur of Bangui (Central African Republic). The quality assurance program of the laboratory has been also involved in the analysis of environmental samples, under the coordination of the WHO Collaborating Centre for *Mycobacterium ulcerans* (Victorian Infectious Diseases Reference Laboratory in Melbourne).

### Detection of acid-fast bacilli in saliva

Positive PCR saliva of *Appasus sp.* were pooled according to the sampling month and diluted in PBS (final volume 160µl). To detect the acid-fast bacilli, smears of suspensions (10µl of saliva suspension pool) were stained by the Ziehl-Neelsen procedure and examined using an oil immersion lens (100×) in an Olympus binocular microscope (model CH30 Olympus) (Figure S1).

### Inoculation of saliva collected from captured *Appasus* into the mouse tail skin

Six week old female BALB/c mice (Charles River France, http://www.criver.com/ico) were maintained under conventional conditions in the animal house facility of the Centre Hospitalier Universitaire, Angers, France (Agreement A 49 007 002), adhering to the institution's guidelines for animal husbandry. From each saliva pool, 3 mice were inoculated subcutaneously with 50 µl of suspension, using 26-gauge needles. The first lesions appeared after the fourth month of inoculation, with inflammatory lesions of the tails of three mice (two mice were inoculated with positive PCR saliva from the April pool, and one mouse from the July pool). Two weeks later, a small oedema was observed and mice were sacrificed. Tissue specimens from mice were weighed, minced with disposable scalpels in a Petri dish and ground with a Potter–Elvehjem homogeniser, size 22, (Kimble/Kontes, Vineland, NJ), in 0.15 M NaCl to obtain a tenfold dilution. Smears of suspensions (10 µl) were stained by the Ziehl Neelsen procedure. DNA was extracted from this material and purified to detect and quantify *M. ulcerans* by quantitative PCR, as described above. Tissue suspensions were decontaminated using an equal volume of *N*-acetyl-L -cysteine sodium hydroxide (2%), as previously described [44], and 0.2 ml of each suspension was inoculated onto two Löwenstein–Jensen slants and incubated at 30°C (Figure S1).

### Statistical analysis

The number of water bugs collected per sampling was modelled using a negative binomial regression, with a random intercept to allow for within-day correlations of samples collected the same day. In this model, the effect of “month of collection” was studied as a fixed effect by introducing dummy variables associated with months of collection. Pearson Chi-square tests were used to compare proportions, and in particular the proportion of insects belonging to a given family (e.g., Belostomatidae, Notonectidae, …) by month of the study, the proportion of insect pools positive for the presence of *M. ulcerans* DNA by month of the study, the proportion of insect pools positive for the presence of *M. ulcerans* DNA by insect family, and the proportion of saliva samples of *Appasus sp.* (Belostomatidae) positive for *M. ulcerans* DNA by month of the study.

## Results

### Taxonomic composition of water bug community

**Figure 2:**
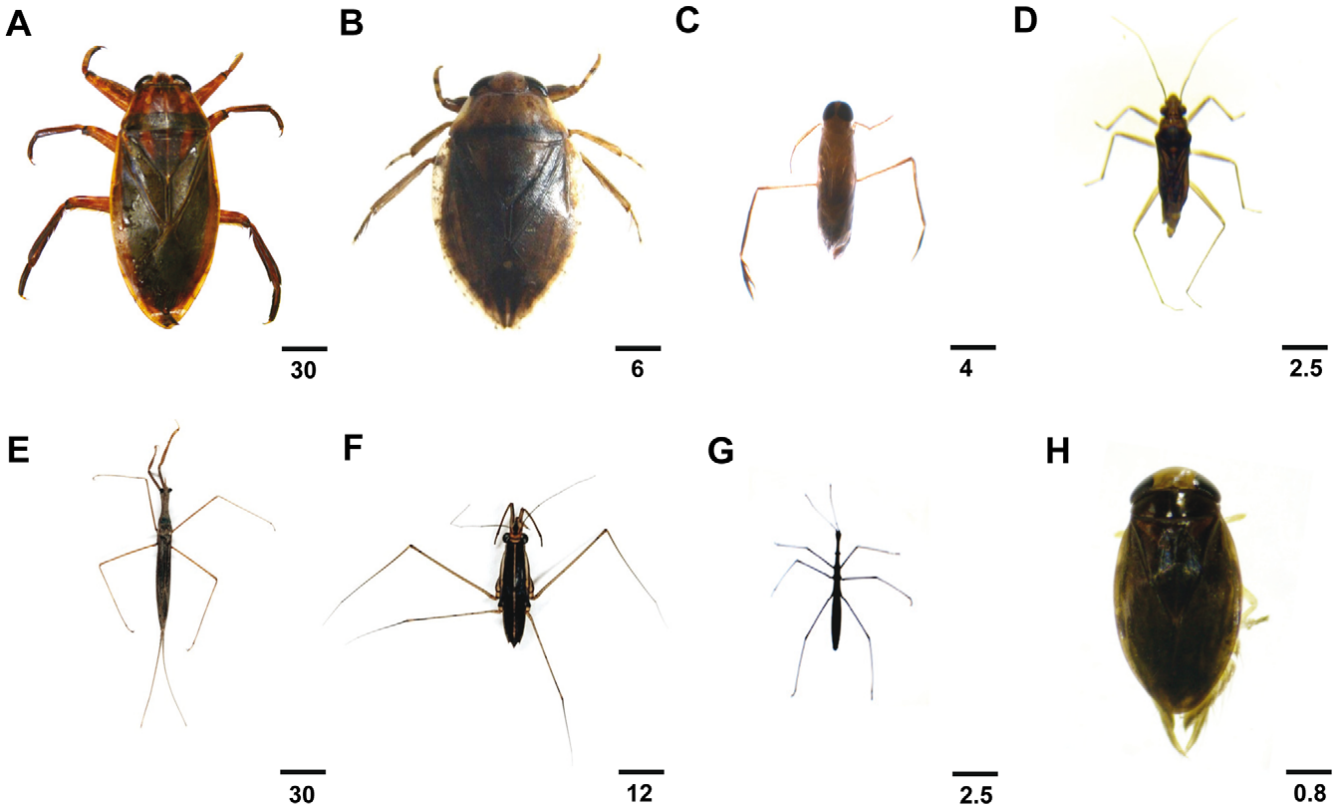
Specimens of aquatic bugs (Hemiptera) collected during the study (dorsal habitus view). Two species of Belostomatidae family: (A) *Appasus sp.*, a biting and flying water bug, (B) *Lethocerus sp.*, the biggest water bug, (C) Notonectinae, biting and flying water bug, (D) Mesoveliidae, a semi-aquatic bug, (E) Ranatrinae, family of Nepidae, a biting water bug, (F) Gerridae, a semi-aquatic bug, (G) Hydrometridae, a semi-aquatic bug, and (H) Micronectinae, family of Corixidae, a fluid feeder water bug. Scale bar: (A) (E) 30 mm, (B) 6 mm, (C) 4 mm, (D) (G) 2.5 mm, (F) 12 mm, (H) 0.8 mm.

The inventory of water bug families in Centre Province of Cameroon was undertaken in Buruli ulcer endemic and non endemic areas, along the Nyong River. Among 7,407 collected specimens, seven aquatic Heteroptera families (Four true aquatic bugs and three semi-aquatic bugs) present in both areas were identified: Belostomatidae, Notonectidae, Nepidae, Corixidae, Gerridae, Mesoveliidae and Hydrometridae (Table 2 and Figure 2). The two most diversified families in our study were the families Notonectidae and Belostomatidae. Seven undetermined morphotypes were present in the Notonectidae family, two belonging to Anisopinae subfamily and five to Notonectinae subfamily. *Appasus sp.*, *Lethocerus* and another unidentified morphotype were present in the Belostomatidae family. Only one subfamily was identified in Nepidae and Corixidae families, respectively Micronectinae and Ranatrinae. All families identified in this study can be characterized as carnivorous predatory fluid-feeders, with the exception of the Corixidae family (plant feeders). Three families (Belostomatidae, Notonectidae, Nepidae), among the six carnivorous ones, are able to bite humans and to fly.

**Table 2:** Identification of aquatic heteroptera collected in endemic and non-endemic areas for Buruli ulcer.

Category	Family	Sub family	Genus	Humans-biting	Endemic site	Non endemic site
True water bugs	Belostomatidae	Belostomatinae	*Appasus*	Yes	+	+
	Belostomatidae	Lethocerinae	*Lethocerus*	Yes	+	+
	Notonectidae	Anisopinae	ND*	Yes	+	+
	Notonectidae	Notonectinae	ND	Yes	+	+
	Nepidae	Ranatrinae	ND	Yes	+	+
	Corixidae	Micronectinae	ND	No	+	+
Semi-aquatic bugs	Gerridae	Gerrinae	ND	?	+	+
	Mesoveliidae		ND	No	+	+
	Hydrometridae		ND	No	+	+

*No determination key available.

### Population dynamics of water bugs in Buruli ulcer endemic area

Cameroon has a tropical climate which varies from equatorial in the South to Sahelian in the North. The equatorial South, where the Buruli ulcer endemic area is located, has two wet seasons and two dry seasons. One wet season occurs between March and June and the main wet season occurs between August and November. One dry season occurs between June and August and the main dry season occurs between December and March. The population dynamics of water bugs was investigated in an endemic area for Buruli Ulcer (district of Akonolinga). In order to get comparable results, insects were captured at periodic intervals by the same operator (with standardized sampling methods), in the same water body each time.

**Figure 3:**
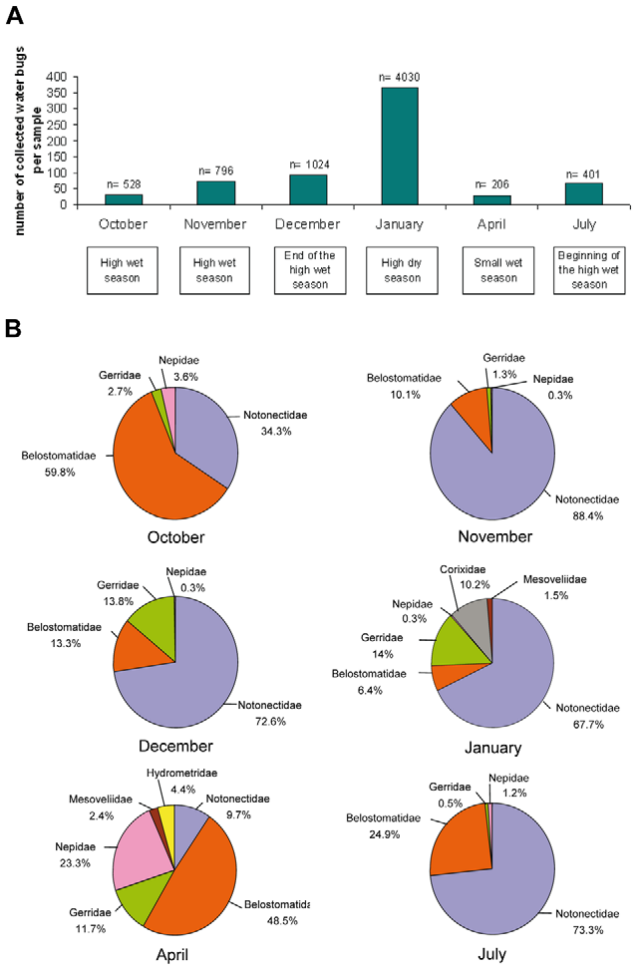
Variation in water bug abundance and taxa distribution in collected samples from an endemic area for Buruli ulcer. (A) Variation in water bug abundance per month, n corresponding to total collected water bugs. (B) Family heteroptera composition and distribution from October to July.

Large fluctuations of water bug density were observed among the samples (Figure 3A). The highest number of collected insects per sampling was recorded in January, during the long dry season (median = 369), whereas in other months, the median number of captured insects per sampling varied between 25 and 94 specimens (*p*<0.001, according to the negative binomial regression model estimating the count of insects per sampling).

With respect to water bug families, the following variations in the sample composition were observed: out of seven families, four families (Belostomatidae, Notonectidae, Gerridae, Nepidae) were collected throughout the study period (Figure 3B), whereas the three other families (Corixidae, Mesoveliidae and Hydrometridae) were found only in January and/or April (Figure 3B). The most abundant family was Notonectidae (67% of total collected water bugs). The proportions for the other families were: 14.2% (Belostomatidae), 10.5% (Gerridae), 5.6% (Corixidae), 1% (Hydrometridae) and 0.1% (Mesoveliidae). The relative abundance of families fluctuated over the year. For example, Belostomatidae and Notonectidae represented respectively 59.8% and 34.3% of total insects in October, and 10.1% and 88.4% in November (Figure 3B). Moreover it was observed that in January Corixidae reached the highest abundance (10.2% out of total water bugs), whereas in January and April the highest water bug diversity was noticed (Figure 3B). All these data suggest that the long dry season corresponds to the period during which highest water bug diversity and abundance occur.

### *M. ulcerans* DNA detection in insect tissues from Buruli ulcer endemic site

Detection of *M. ulcerans* in samples collected in Buruli ulcer endemic and non endemic sites was performed by PCR targeting the IS*2404* insertion and the KR domain which encodes a polyketide synthase.

**Figure 4:**
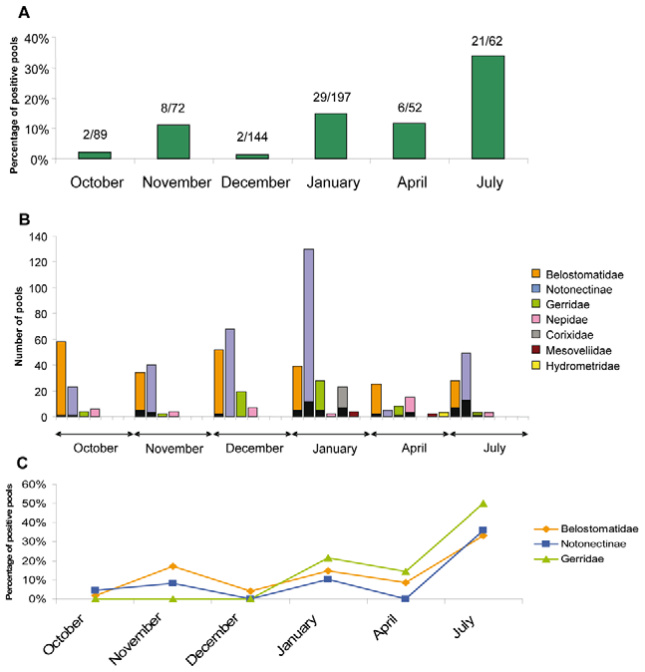
Detection of *M. ulcerans* DNA in insect tissues. The analysis was performed on 616 insect pools corresponding to 3647 individual specimens. (A) Percentage of positive pools over the period of study, (n) corresponding to the number of positive pools out of total pools. (B) Number of pools and number of positive pools (black part of bars represent the number of positive PCR pools) following water bug families and period of collection. (C) Monthly trends in *M. ulcerans* DNA positivity rate by family.

From 3647 water bugs collected in the endemic area, 68 pools out of 616 (11%) were positive for both markers, IS*2404* and Ketoreductase (Figure 4A). In addition *M. ulcerans* DNA was detected in five out of seven analyzed insect families. The rate of colonization in these pools was around 10%, except for the Corixidae family (Micronectinae) captured only in January, for which the rate reached 43.7% (*p* = 0.008, Pearson Chi-square test) (Figure 4B). Of note, this result was confirmed for individual Corixidae specimens (n = 72). However, given the very low number of collected water bugs from Mesoveliidae and Hydrometridae families (33 and 9 specimens, respectively), it is difficult to draw clear conclusions, in this case, about their possible subversion as hosts for *M. ulcerans*.

The rate of insect colonization by *M. ulcerans* fluctuated between 1.4% and 33.9% according to the sampling period, with a peak in July (33.9%) (*p* = 0.008, Pearson Chi-square test) (Figure 4A). Moreover, in the present study, no correlation was established between abundance of water bugs and rate of colonization of water bugs by *M. ulcerans*. All families -notably Belostomatidae, Notonectinae and Gerridae- displayed very similar fluctuations in colonisation rates by *M. ulcerans* (Figure 4C), with the exception of Nepidae (for which the sample size was limited).

### Detection of *M. ulcerans* DNA in insect tissues from Buruli ulcer non endemic site

From 422 water bugs caught in a Buruli ulcer non endemic area, no pool out of 80 was found positive (Table 3). Significantly, in these same April and July periods, 11.5% and 33.9%, respectively, pools were found positive in the endemic site situated 100 kms away.

**Table 3:** Detection of *M. ulcerans* DNA in aquatic heteroptera tissues collected in Buruli ulcer endemic and non-endemic areas.

		Endemic site			Non endemic site		
			Pools			Pools	
	Family	No. of water bugs	No.	Positive (%)	No. of water bugs	No.	Positive (%)
	Belostomatidae	100	23	2 (8.7)	21	5	0
	Notonectidae	20	5	0	17	3	0
	Nepidae	48	12	3 (25)	95	19	0
**April**	Corixidae	0	0	-	0	0	-
	Gerridae	24	7	1 (14.3)	0	0	-
	Mesoveliidae	5	2	0	0	0	-
	Hydrometridae	9	3	0	0	0	-
	Belostomatidae	100	21	7 (33.3)	14	5	0
	Notonectidae	294	36	13 (36.1)	32	9	0
	Nepidae	5	3	0	0	0	-
**July**	Corixidae	0	0	-	44	4	0
	Gerridae	2	2	1 (50)	24	5	0
	Mesoveliidae	0	0	-	6	3	0
	Hydrometridae	0	0	-	10	3	0
**Total**		607	114	27 (16.8)	422	80	0

### Detection of *M. ulcerans* in the saliva collected from *Appasus sp.* sampled in Buruli ulcer endemic area

**Figure 5:**
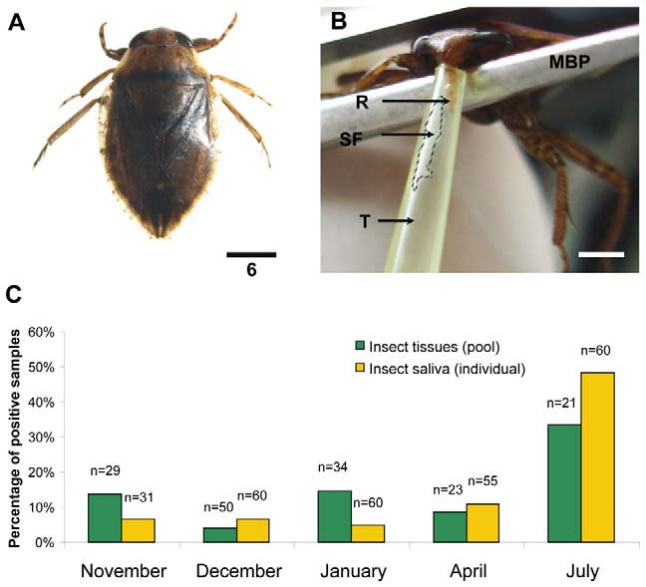
Detection of *M. ulcerans* DNA in tissues and in saliva of *Appasus sp.* (Belostomatidae). (A) Detection of *M. ulcerans* DNA in saliva was performed on *Appasus sp.* (Scale: 6 mm). (B) Water bug was grasped with metallic blunt pincers (MBP). The saliva was collected by introduction of rostrum (R) in a tip (T). The white saliva fluid (SF) (dotted line) could be observed. (C) Detection of *M. ulcerans* DNA in saliva was performed on saliva samples and on insect tissues over the period of study.

Only living Belostomatidae insects of the genus *Appasus sp.* (Figure 5A and B) were allowed to salivate, for technical reasons. The saliva of these insects was first monitored for the presence of *M. ulcerans* DNA (Figure 5A and B). The individual saliva samples analysed by IS*2404* and KR PCR were found positive in 51/293 of cases (17.4%), with a peak in July. Similar patterns were observed with homogenate tissue of *Appasus sp.* (Figure 5C). Interestingly, few acid-fast bacilli were observed in saliva samples of three individual positive pools (November, April and July). Their viability was evaluated by inoculation of PCR positive saliva into the tails of 21 Balb/c mice. Using quantitative PCR, quantity of inoculated bacilli was determined to range between 1×10^2^ and 5×10^3^ bacilli per ml. Four months after the subcutaneous injection, three mice displayed lesions typical of *M. ulcerans*, in which acid-fast bacilli were detected. Quantity of bacilli was estimated by quantitative PCR to range between 6×10^4^ and 3×10^5^ bacilli per ml of grounded tissue. This result suggests growth of *Mycobacterium ulcerans* in mouse tail. It can be noticed, however, that conventional methods failed to isolate the bacilli by culture from mouse tissues presenting clinical lesions.

## Discussion

The mode of transmission of *M. ulcerans* to humans remains unclear, with several different mechanisms proposed in the last few decades. Within the WHO, a consensus therefore emerged to investigate the processes underlying i) the transmission of *M. ulcerans* to humans and ii) the dissemination of *M. ulcerans* in the environment.

### Objectives and rationale of the study

The aim of this study was to assess the role of water bugs as hosts and vectors of *M. ulcerans*, in the complete environmental context. To this end, we carried out an extensive field study on unprecedented temporal and spatial scales, monitoring the distribution of water bugs harboring *M. ulcerans* and the dissemination of *M. ulcerans* in the environment. We assessed water bug diversity and determined the frequency of insect tissue colonization by *M. ulcerans* in the various seasons, in an area in which Buruli ulcer is endemic (Akonolinga, Cameroon). For the purposes of comparison, the study also covered an area in which Buruli ulcer is not endemic. In short, the specificities of the study are: (i) focusing entirely on water bugs; (ii) the collection of samples from the same water body in all four seasons and (iii) large-scale sample collection in Buruli ulcer endemic and non endemic areas, with subsequent analysis of the captured water bug specimens (7407 specimens collected, 696 pools analyzed).

### Inventory of water bugs and fluctuations in their density

We document here, for the first time, fluctuations in the density of water bug families in an area in which Buruli ulcer is endemic, over the course of a year. The highest density of water bugs is recorded in January, during the long dry season. Variations of water bug density described here are in agreement with those in another study in a tropical area (Costa Rica) [45]. The causes of these fluctuations remain unclear, but several possible factors have been identified, including abrupt changes in environmental conditions or prey density [33], [45]–[46].

Seven water bug families were identified in Buruli ulcer endemic and non endemic areas, including many unknown species. This latter point is not surprising, as determination keys for water bug species are not yet available for West Africa. One key finding of our study was the striking difference between the endemic and non endemic areas in terms of the density of water bugs, with the density in the endemic area more than 10 times higher that in the non endemic area. It can be noticed that this observation is not in accordance with results in another published study [31]. However, the conclusions of this previous study were drawn based on data for a significantly smaller number of specimens (only 200 water bugs; 2% of the invertebrates collected [31]).

### Water bugs as hosts for *M. ulcerans*

*M. ulcerans* DNA was detected in five of the seven families of water bugs in the endemic area. The mean rate of colonization was about 10%. However, large fluctuations were observed in the rate of insect colonization by *M. ulcerans* (1.4 to 33.9%). As observed for other hosts of microorganisms [47]–[48], there was no correlation between water bug density and rates of water bug colonization by *M. ulcerans*. We detected no *M. ulcerans* DNA in insects from the non endemic area, despite the high rates of colonization reported for the nearby endemic area.

One key difference between the endemic and non endemic areas concerned human activity. Despite their close physical proximity, the non endemic area was largely unaffected by human activity, whereas, in the endemic area, the bank of the Nyong River had been deforested, for agricultural and fishing activities (Figure 1B and C). Interventions disrupting the environment have been identified, in several studies, as factors potentially favoring the establishment of *M. ulcerans* in remodeled environments [13]–[15], [49].

The observed fluctuations in colonization rate may be accounted for by changes in the level of water in the Nyong River, which falls markedly in the dry season. Our results are supported by the findings of an epidemiological study performed in 1993 [50], in which low water levels in the dry season were found to favor the transmission of *M. ulcerans* to humans. Several factors may be involved in this phenomenon, including greater access to nutrients (resulting in an increase in *M. ulcerans* density) and the abundance of aquatic vegetation favoring *M. ulcerans* biofilm formation [26], [30], [51] (increasing the level of contact between *M. ulcerans* and water bugs).

The water bugs from the family Corixidae were the only phytophagous insects inventoried here. These water bugs were detected only in January, when they were present in high abundance, and displayed the highest rate of colonization by *M. ulcerans* (43.7%). The high frequency of colonization by *M. ulcerans* in Corixidae supports the hypothesis that aquatic plants may be the primary reservoir of *M. ulcerans*, as previously suggested [26]. This specific family may be involved in spreading the bacillus to other trophic levels, as they are eaten by other water bugs, aquatic invertebrates and vertebrates. These observations suggest that *M. ulcerans* may colonize different levels within the trophic chain (aquatic plants, invertebrates and vertebrates), as already considered [21], [30], [52]–[53]. In addition, three water bug families are known to be good flyers [33], [45], and were identified as *M. ulcerans* hosts in our study. These water bugs may therefore be involved in disseminating *M. ulcerans* in the environment, as previously proposed by Portaels and Meyers [34].

### Water bugs as vectors for *M. ulcerans*

We showed that water bug saliva could harbor bacilli. *M. ulcerans* may therefore be present in the saliva under natural conditions, with the bacilli colonizing the salivary glands of the insect. This could provide a route for *M. ulcerans* transmission in natural conditions, in accordance with previous experimental demonstrations in laboratory conditions [24], [28]. It should be noted that the bacilli present in the saliva of *Appasus sp.* induced an *M. ulcerans*-containing lesion following the inoculation of mouse tail. However, it was not possible to isolate these bacilli by conventional culture methods. The lack of appropriate culture media and decontamination procedures adapted to the isolation of *M. ulcerans* (from the environment) thus remain a major handicap, hindering investigations of the ecology and mode of transmission of this mycobacterium.

### Highlights, difficulties and perspectives

The various results presented above provide further evidence that water bugs are hosts and vectors of *M. ulcerans*, and provide insight into the environmental context underlying transmission. However, no definitive conclusion can yet be drawn concerning the precise importance of this route of transmission. The presence, in human sera, of antibodies binding water bug salivary gland extracts may be accounted for by the exposure of humans to water bug bites [27]. Indeed, reports of the exposure of humans to blood-feeding arthropods, which are known to act as hosts and vectors for parasitic microorganisms, are becoming increasingly frequent [38]–[39].

To gain a complete picture of the transmission route it would have been desirable to explore the relationship between the incidence of the disease in humans and the rate of colonization of water bugs by *M. ulcerans*, over a one-year period. Such exploration was however hampered by the amount of accessible information, with several important parameters not available : (i) incubation time between exposure to *M. ulcerans* and the appearance of the first clinical lesions (currently estimated at between a few weeks and several months); (ii) the slow progression of clinical lesions, resulting in patients being diagnosed at different stages of the disease (rarely at early stages) and (iii) the small number of Buruli ulcer patients diagnosed with early lesions between October 2007 and July 2008 in Akonolinga (84 Buruli ulcer patients, including 15 with early lesions).

In conclusion, this study sheds light on the natural history of *M. ulcerans* within its ecosystem. The observed fluctuations in insect colonization rates suggest that there may be a particularly favorable period for the development of *M. ulcerans* in natural conditions, and a favorable period for the transmission of *M. ulcerans* to humans (as previously suggested [50], [58] and observed for *Plasmodium sp.* [47], [59]–[60]). It is also noticeable that the results here can be put to advantage for practical applications, with surveillance and prevention purposes [27], [61]. More precisely, our work suggests that the detection of *M. ulcerans* in water bug saliva could be used as an environmental indicator of the risk of *M. ulcerans* transmission to humans. It would then be possible to set up environmental surveillance (detection of *M. ulcerans* DNA in water bug tissue and saliva) in non endemic areas close to Buruli ulcer endemic areas. Health messages concerning environmental risk factors could be specifically targeted at populations newly exposed to the risk of *M. ulcerans* infection, as is already the case for protective factors (wearing long clothing during farming activities and use of bed nets) [16].

## Supporting Information

Figure S1 Main steps followed to detect *M. ulcerans* in water bug saliva.
(0.10 MB TIF)
