# Supplementary figures and images for: Seasonal and Regional Dynamics of M. ulcerans Transmission in Environmental Context: Deciphering the Role of Water Bugs as Hosts and Vectors
Source: PLoS Negl Trop Dis. 2010 Jul 6;4(7):e731. doi: 10.1371/journal.pntd.0000731 (PMC2897839; doi:10.1371/journal.pntd.0000731)

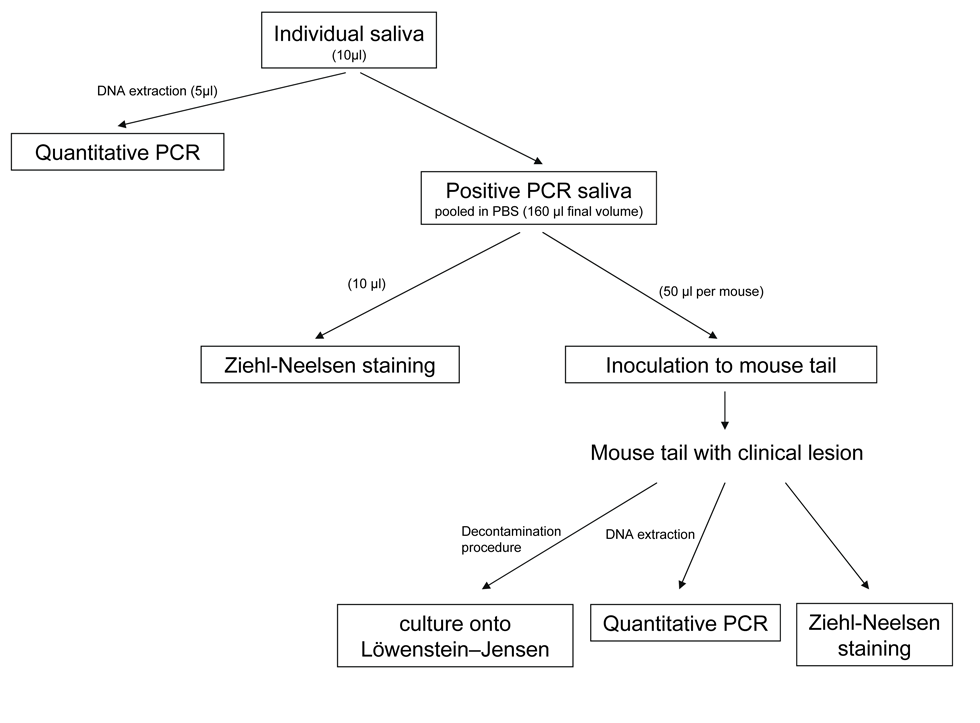

Supplement: Figure S1 — Main steps followed to detect M. ulcerans in water bug saliva. (0.10 MB TIF) [file pntd.0000731.s001.tif]
